# Effects of annual influenza vaccination on morbidity and mortality in patients with Systemic Lupus Erythematosus: A Nationwide Cohort Study

**DOI:** 10.1038/srep37817

**Published:** 2016-12-02

**Authors:** Chi-Ching Chang, Yu-Sheng Chang, Wei-Sheng Chen, Yi-Hsuan Chen, Jin-Hua Chen

**Affiliations:** 1 Division of Allergy, Immunology and Rheumatology, Department of Internal Medicine, Taipei Medical University Hospital, Taipei, Taiwan; 2 Division of Allergy, Immunology and Rheumatology, Department of Internal Medicine, School of Medicine, College of Medicine, Taipei Medical University, Taipei, Taiwan; 3 Division of Allergy, Immunology, and Rheumatology, Department of Internal Medicine, Shuang Ho Hospital, Taipei Medical University, New Taipei City, Taiwan; 4 Division of Allergy, Immunology, and Rheumatology, Department of Internal Medicine, Taipei Veterans General Hospital, and National Yang-Ming University, Taipei, Taiwan; 5 Biostatistics Center, College of Management, Taipei Medical University, Taipei, Taiwan; 6 Biostatistics Center and School of Health Care Administration, College of Management, Taipei Medical University, Taipei, Taiwan

## Abstract

Studies on the clinical efficacy of influenza vaccination on patients with systemic lupus erythematosus (SLE) are scant. The present study compared the incidence of hospitalization, morbidity, and mortality in patients with SLE between cohorts with and without influenza vaccination. We used the Taiwan’s insurance claims data between 2001 and 2012 for identifying annual adult patients with SLE with (N = 1765) and without (N = 8360) influenza vaccination. The incidence rate ratio and hazard ratio (HR) for morbidities and mortality were measured for the vaccine and nonvaccine cohorts. The vaccine cohort had a lower hospitalization rate than did the nonvaccine cohort, with an adjusted HR of 0.82 (95% CI 0.73–0.92). Furthermore, the vaccine cohort was less likely to be admitted to the intensive care unit [adjusted HR 0.55 (95% CI 0.39–0.79)], to be hospitalized for septicemia, bacteremia, or viremia [adjusted HR 0.48 (95% CI 0.32–0.73)], to undergo in-hospital dialysis [adjusted HR 0.40 (95% CI 0.20–0.81)], and were less predisposed to death [adjusted HR 0.41 (95% CI 0.27–0.61)]. In conclusion, influenza vaccination in patients with SLE is associated with a reduced risk of morbidity and mortality.

Systemic lupus erythematosus (SLE) is an autoimmune disease characterized by relapse and remittance. At least 50% of the patients experience one episode of severe infection caused by common or opportunistic microorganisms^1^,^2^. Infections cause 20–55% deaths in patients with SLE^3^. Influenza, one of the most frequent infections, is estimated to annually infect 5% of the adult population^4^. In the Unites States, influenza causes more than 225,000 hospitalizations and 36,000 deaths annually^5^,^6^. Morbidity and mortality caused by influenza increase in elderly people, immunocompromised patients, and patients with chronic diseases^7^. Vaccination substantially aids in preventing influenza-related morbidity and mortality and is recommended for immunocompromised patients^8^. Because influenza vaccination does not induce disease activity in SLE, annual influenza vaccination for patients with SLE is highly recommended^9^,^10^.

However, whether vaccination is effective in patients with SLE is unclear, as patients with SLE have decreased primary and secondary immune responses^11^. Moreover, immunosuppressive drugs may further decrease immune response following vaccination. Studies on the effect of influenza vaccination on patients with SLE^12^,^13^,^14^,^15^,^16^,^17^,^18^,^19^,^20^ have suggested the safety of vaccination but with reduced immunogenicity, which is attributed to the immunosuppressive therapy. Holvast *et al*. reported a decreased antibody response in patients with SLE. Seroprotection (titre ≥40) rates were lower in patients with SLE than in healthy adults, limiting clinical protection from influenza in some vaccinated patients^10^. By contrast, Kanakoudi *et al*. reported normal efficacy levels of vaccination^21^. Furthermore, whether vaccination in patients with SLE protects them throughout the influenza season, that is, whether protective antiinfluenza titres increased following vaccination, is clinically relevant but unclear. Studies assessing the clinical efficacy of the influenza vaccination on patients with SLE are lacking. Therefore, this study evaluated the efficacy of influenza vaccination for reducing morbidity and mortality in patients with SLE through a population-based cohort study.

## Methods

### Data source

The study utilized the National Health Insurance Research Database (NHIRD) of Taiwan, which records inpatient and ambulatory care claims from 2001 to 2012. The National Health Insurance (NHI) program of the Bureau of National Health Insurance (BNHI) covers >98% of the Taiwanese population. It utilizes a comprehensive and computerized database that records all medical claims for ambulatory care services and hospitalization, facilitating a nationwide population-based cohort study. The BNHI routinely validates diagnoses by reviewing the original medical charts of patients. The NHIRD has established a registry system for catastrophic illnesses, including SLE. The completeness and accuracy of the NHI claims databases have been assured by the aforementioned agencies. The study was approved by the Institutional Review Board of Taipei Medical University (approval number: N201509007). The study was carried out in accordance with the approved guidelines. Informed consent of the study participants was not required because the dataset used in this study consists of de-identified secondary data released for research purposes.

### Retrospective cohort study

This is a retrospective cohort study that used the national database in Taiwan. All patients with SLE, identified using the International Classification of Diseases, Ninth Revision, Clinical Modification (ICD9-CM) code 710.0 for catastrophic illnesses, in the registry during 2001–2011 were enrolled in the study. Since the actual application date was unable to found before 2001, we enrolled the SLE patients who applying the registry for catastrophic illnesses after 2001. The date of the first ambulatory care visit with a diagnosis of SLE (age ≥18) was set as the index date for the study cohort. To identify new SLE cases, patients with an index date before January 1, 2001, were excluded. Patients with SLE who completed seasonal influenza vaccination (ICD-9-CM V04.7 and V04.8) formed the vaccine cohort, with the date of vaccination defined as the index date for measuring the follow-up period. Patients with SLE who received pneumococcal vaccine were excluded. In addition, patients newly diagnosed with SLE without both annual influenza and pneumococcal vaccinations formed the nonvaccine cohort. The index date of the nonvaccine cohort was selected randomly from the same date of the vaccine cohort with the same application year. The corresponding vaccine cohort was selected randomly after matching for index month.

### Study outcomes

The follow-up person-year for each participant was measured from the index date to 365 days or until censure because of death or withdrawal from the insurance system. The outcome events included total hospitalization; hospitalization for pneumonia (ICD-9-CM 480–487), septicemia, bacteremia, viremia (ICD-9-CM 038.x, 790.7, and 790.8), and heart disease (ICD-9-CM 401–429); intensive care unit (ICU) admission; in-hospital dialysis; and death.

### Covariate assessment

For ascertaining other relevant comorbidities, we included coronary artery disease (ICD-9-CM 410–413, 414.01–414.05, 414.8, and 414.9), congestive heart failure (ICD-9-CM 428, 398.91, 402.x1), hypertension (ICD-9-CM 401–405), hyperlipidemia (ICD-9-CM 272), atrial fibrillation (ICD-9-CM 427.31), chronic obstructive pulmonary disease (ICD-9-CM 490–496), renal disease, cancer (ICD-9-CM 140–149, 150–159, 160–165, 170–175, 179–199, 200, 202, 203, 210–213, 215–229, 235–239, 654.1, 654.10, 654.11, 654.12, 654.13, 654.14), chronic hepatitis (ICD-9-CM 571, 572.2, 572.3, 572.8, 573.1, 573.2, 573.3, 573.8, 573.9), and stroke (ICD-9-CM 430–438). If these diagnostic codes were present in two or more ambulatory claims 1 year before the index date, they were recorded as comorbidities. These comorbidities were tested in univariate analysis and were adjusted for in multivariate Cox regression analysis.

### Statistical analysis

We compared the demographic status (i.e., age, sex) and comorbidities, namely coronary artery disease, congestive heart failure, hypertension, hyperlipidemia, atrial fibrillation, chronic obstructive pulmonary disease, renal disease, cancer, chronic hepatitis, stroke, dementia, and rehabilitation, between the vaccine and nonvaccine cohorts. The differences were examined using the *χ*^2^ test for categorical variables and the *t*-test for continuous variables. If the distribution of the steroid dose was abnormal, data were analyzed using the Wilcoxon rank sum test. The follow-up duration was used to estimate the incidence rates of hospitalization, pneumonia or influenza, respiratory failure, ICU admission, and mortality. The Cox proportional hazards regression model was used to estimate the corresponding hazard ratios (HRs) and 95% confidence intervals (CIs). The HRs were adjusted for sex, age, and the aforementioned comorbidities in the Cox model. Patient data were stratified by age (<65 years and ≥65 years) and analyzed. In addition, the Cox proportional hazard model was used to estimate the baseline cumulative hazard rate for various outcomes in the vaccine and nonvaccine cohorts. SAS version 9.3 (SAS Institute, Cary, NC, USA) was used for all data analyses. P < 0.05 was considered statistically significant.

## Results

12,728 patients with SLE with catastrophic illness certification were identified from 2001 to 2011, and 10,125 adults with SLE were enrolled after the following patients were excluded: 214 patients with missing age or sex data; 827 patients who died before influenza vaccination, 1166 patients aged <18 years, and 111 patients who received pneumococcal vaccine. Table 1 presents the annual proportion of influenza vaccination in newly diagnosed SLE. The vaccine and nonvaccine cohorts comprised 1765 and 8360 newly diagnosed patients with SLE with and without vaccination for influenza, respectively. When stratified by age, elderly patients (≥65 years) constituted most of the vaccinated patients. Table 2 presents the baseline characteristics. The female to male ratio (female predominance) did not differ significantly between the two cohorts. The mean age in the vaccine cohort (46.23 ± 17.69 years) was substantially higher than that in the nonvaccine cohort (37.27 ± 13.21 years). Patients in the vaccine cohort had more comorbidities than those in the nonvaccine cohort. The median of the steroid dose was identical in the cohorts.

Regardless of morbidities, the IRR for morbidity in the vaccinated and nonvaccinated cohorts did not differ (Table 3). In the Cox proportional hazards analysis adjusting for various factors (age, sex, and comorbidities), the adjusted HRs revealed a protective association with the vaccination. The total hospitalization rate was lower in the vaccine cohort (adjusted HR 0.82 [95% CI 0.73–0.92]) than in the nonvaccine cohort (Table 3). Compared with patients in the nonvaccine cohort, those in the vaccine cohort were less likely to be admitted to the ICU [adjusted HR 0.55 (95% CI 0.39–0.79)]. In addition, patients in the vaccine cohort were less likely to be hospitalized for septicemia, bacteremia, or viremia [adjusted HR 0.48 (95% CI 0.32–0.73)]; less likely to undergo in-hospital dialysis [adjusted HR 0.40 (95% CI 0.20–0.81)]; and were less predisposed to death [adjusted HR 0.39 (95% CI 0.26–0.59)]. In Table 4, the age-stratified analysis revealed that the vaccine to nonvaccine cohort rate of total hospitalization, hospitalization for pneumonia, ICU admission, and death reduced with age.

**Figure 1:**
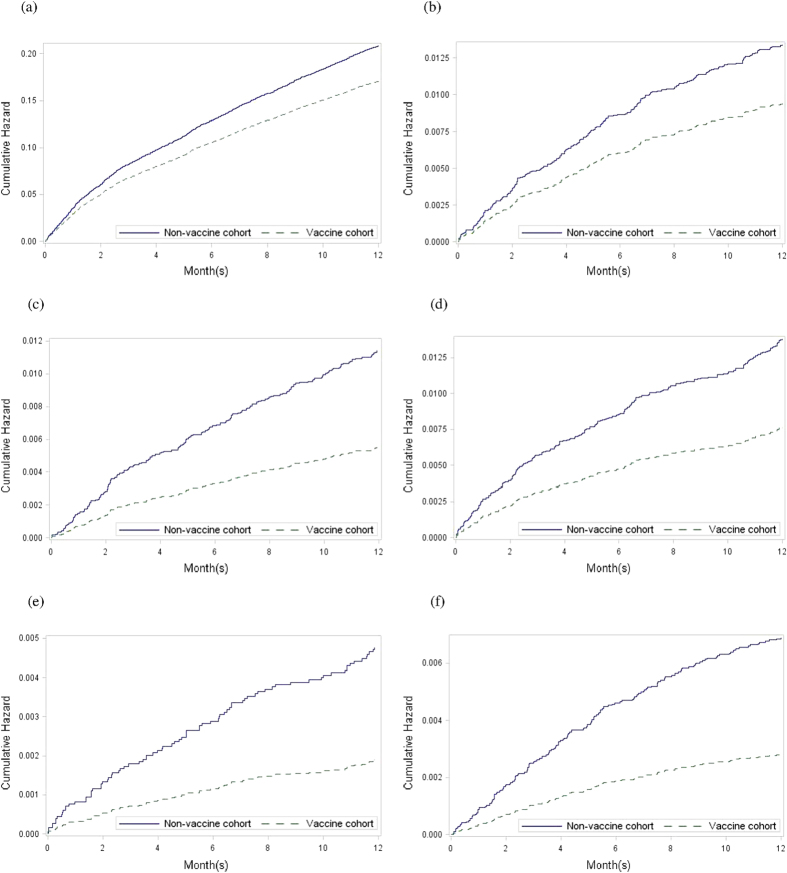
Estimated baseline cumulative hazard rate for different outcomes under the Cox proportional hazard model in patients with SLE with and without vaccination (**a**) Total hospitalization (**b**) Hospitalization for pneumonia (**c**) Hospitalization for septicemia, bacteremia, or viremia (**d**) Intensive care unit admission (**e**) In-hospital dialysis (**f**) Death.

Compared with the nonvaccine cohort, the vaccine cohort had significantly lower rates of cumulative proportion of total hospitalization (P = 0.01, Fig. 1a); hospitalization for pneumonia (P = 0.03, Fig. 1b); hospitalization for septicemia, bacteremia, or viremia (P = 0.0002, Fig. 1c); ICU admission (P < 0.0001, Fig. 1d); in-hospital dialysis (P < 0.0001, Fig. 1e); and mortality (P < 0.0001, Fig. 1f).

## Discussion

This nationwide population-based study demonstrated that influenza vaccination in adults with SLE was associated with low morbidities, including total hospitalization, ICU admission, hospitalization for pneumonia (≥65 years old); hospitalization for septicemia, bacteremia, or viremia; and in-hospital dialysis. In addition, influenza vaccination was associated with a 49% lower mortality risk. This is the first study addressing clinical outcomes of vaccination in patients with SLE.

Influenza infection-related morbidity and mortality increase in immunocompromised patients^8^. Because of high annual incidence of influenza, which affects 5–20% of the general population^22^, vaccination is a clinically relevant concern in patients with SLE. Influenza vaccination in patients with SLE is safe because it does not induce disease activity^23^. Our study demonstrated that influenza vaccination in patients with SLE is associated with a reduced risk of morbidity and mortality, supporting the annual vaccination in such patients, as recommended by Gluck *et al*.^24^.

Patients with SLE have a high influenza infection risk of infection. Low complement levels, functional alterations of phagocytic cells, impaired cellular immunity with lymphopenia and decreased cytokine production, decreased immunoglobulin production, low microorganism eliminating capacity by the reticulo–endothelial system, and immunosupressant drugs are among the predisposing factors for the increased infection rate^1^,^2^. Influenza increases the risk of pneumonia and bacterial pneumonia, and vaccination is a cost-effective and efficient means to prevent influenza. Yearly vaccinations are recommended because of the risk of severe influenza in patients with SLE^25^,^26^. Our study revealed that vaccinated elderly patients (≥65 years) with SLE had a low risk of hospitalization for pneumonia.

Several studies have shown that patients with SLE have a reduced antibody response after vaccination compared with healthy adults^14^,^20^,^27^. By contrast, several studies have suggested the efficacy of vaccination in patients with SLE^12^,^13^,^21^. In addition, immunosuppressive drugs may further reduce the immune response following vaccination^28^,^29^,^30^. Whether vaccination is effective in patients with SLE is unclear. However, a favorable clinical outcome of vaccination in patients with SLE was observed in this study. Because vaccinations effectively reduce hospitalizations and deaths in patients with SLE, healthcare providers must ensure that patients receive yearly influenza vaccines.

Since 1998, the Taiwan NHI program provides influenza vaccinations to high-risk patients, including those with SLE and the elderly patients; majority of the vaccinated patients annually receive this service between October 1 and December 31. However, the influenza vaccination rate in this study was low for patients with incident SLE, particularly for those younger than 65 years. The low vaccination rate may be due to the lack of awareness, fear of adverse reactions, and the lack of physician recommendations. In addition, rheumatologists’ workload may be a factor for low vaccination rates. A Canadian study reported that physicians with heavy workloads do not adequately address disease prevention^31^. Furthermore, physicians in the United States do not recommend influenza vaccination to elderly and high-risk patients^32^. In the health care settings of Taiwan, in-patient waiting duration is long and physicians are pressured to shorten the consultations. Physicians treating patients with several chronic conditions discuss and treat each condition, leaving them with less time to introduce a new concern or discuss disease prevention. Furthermore, a lack of knowledge regarding the vitality and usefulness of vaccination may contribute to the low vaccination rate^33^,^34^. These factors are conjectures and must be substantiated.

Studies assessing the clinical efficacy of influenza vaccination on patients with SLE are lacking. Two small studies have demonstrated low infection rates after influenza vaccination in patients with SLE and rheumatoid arthritis and in children with rheumatic diseases^21^,^35^. Liao *et al*. suggest with consideration of a higher risk of SLE exacerbation and a more severe course of infection among SLE patients, influenza vaccination should be promoted among with a low- to –moderate SLE disease activity index score or stable disease^36^. Murdaca *et al*. report that influenza vaccination in SLE patients are (1) efficacious, even if specific immune responses may be lower than in the general population, as generally the humoral response fulfills the criteria for vaccine immunogenicity and (2) safe in inactive disease although may favor a transient increase in autoantibody levels and rarely disease flares^37^. Although there are concerns that immunization may cause SLE exacerbation, evidence from prospective trials suggests that inactivated influenza vaccine are probably safe in patients with stable or inactive SLE disease activity^38^. Indeed, in immunocompromised patients, influenza vaccination benefits (reduced morbidities and mortalities) have been demonstrated^7^.

The strengths of our study include the large representative sample size of adults with SLE, cohort design, and the one-year follow-up for each cohort. The one-year follow-up can determine the possibility of delayed complications of influenza.

The study has several limitations. First, the NHID provided limited information on sociodemographic characteristics, marital status, educational level, smoking habits, and body mass index, and laboratory data were unavailable. These variables were not adjusted for in the analysis. Additionally, information on SLE severity scale, such as disease activity, organ damage and the levels of autoantibodies was unavailable in our data. The lack of drug data, such as immunosuppresives and glucocorticosteroids to adjust for the outcomes of interest could be another limitation. Furthermore, receiving vaccination may be affected by the socioeconomic status and the availability of health care and medical providers. Although multivariate analysis was used, selection bias may have occurred.

In conclusion, influenza vaccination is associated with a reduced risk of hospitalization, pneumonia, ICU admission, in-hospital dialysis, and death. Additional large-scale prospective studies are warranted to investigate the efficacy of influenza vaccination efficacy in adults with SLE.

## Additional Information

**How to cite this article**: Chang, C.-C. *et al*. Effects of annual influenza vaccination on morbidity and mortality in patients with Systemic Lupus Erythematosus: A Nationwide Cohort Study. *Sci. Rep.* **6**, 37817; doi: 10.1038/srep37817 (2016).

**Publisher's note:** Springer Nature remains neutral with regard to jurisdictional claims in published maps and institutional affiliations.

## Figures and Tables

**Table 1:** Proportion of influenza vaccination in patients with SLE in different age groups.

Age Group (y)	N	Influenza Vaccination		Proportion of Vaccination (%)
		Yes	No	
18–29	3401	406	2995	11.94
30–39	2583	370	2213	14.32
40–49	1981	273	1708	13.78
50–64	1516	356	1160	23.48
65–74	448	257	191	57.37
75–84	182	96	86	52.75
85–99	14	7	7	50.00

**Table 2:** Baseline characteristics of patients with SLE with and without vaccination.

Group	Nonvaccine cohort (N = 8360)		Vaccine cohort (N = 1765)		P value*
Variables	n (%)		n (%)		
Gender					0.043
Male	943	(11.28)	229	(12.97)	
Female	7417	(88.72)	1536	(87.03)	
Age (y), Mean (SD)	37.27	(13.21)	46.23	(17.69)	<0.001^a^
Age group					<0.001
18–29	2995	(35.83)	406	(23.00)	
30–49	3921	(46.90)	643	(36.43)	
50–64	1160	(13.88)	356	(20.17)	
≥65	284	(3.40)	360	(20.40)	
1-year steroid dose before index day, median (IQR)	1540	(2881.09)	1570	(2510.00)	0.478^b^
Baseline comorbidity					
Coronary artery disease	253	(3.03)	149	(8.44)	<0.001
Congestive heart failure	215	(2.57)	85	(4.82)	<0.001
Cancer	1091	(13.05)	312	(17.68)	<0.001
Diabetes mellitus	356	(4.26)	135	(7.65)	<0.001
Hyperlipidemia	676	(8.09)	186	(10.54)	0.001
Hypertension	1409	(16.85)	514	(29.12)	<0.001
Atrial fibrillation	28	(0.33)	21	(1.19)	<0.001
Chronic Hepatitis	774	(9.26)	213	(12.07)	<0.001
Stroke	267	(3.19)	133	(7.54)	<0.001
COPD	608	(7.27)	240	(13.26)	<0.001
Chronic Kidney Disease	349	(4.17)	111	(6.29)	<0.001

Abbreviations: COPD, chronic obstructive pulmonary disease.

^*^P values were calculated using the chi-squared test for categorical variables.

^a^P values were calculated using the t test.

^b^P values were calculated using the Wilcoxon rank sum test.

**Table 3:** Comparing the risk of interested outcomes between patients with SLE with and without vaccination.

Group	Nonvaccine cohort			Vaccine cohort			IRR	Crude HR	(95% CI)	Adjusted HR^b^	(95% CI)
Outcomes	n (%)		Incidence^a^	n (%)		Incidence^a^					
Total hospitalization	1894	(22.66)	26.49	405	(22.95)	26.47	1.00	1.00	(0.90–1.11)	0.82	(0.73–0.92)***
Hospitalization for pneumonia	174	(2.08)	2.13	47	(2.66)	2.73	1.28	1.28	(0.93–1.77)	0.70	(0.49–1.00)
Hospitalization for septicemia, bacteremia, or viremia	161	(1.93)	1.96	33	(1.87)	1.90	0.97	0.97	(0.67–1.41)	0.48	(0.32–0.73)***
Hospitalization for heart disease	480	(5.74)	5.99	150	(8.50)	8.96	1.50	1.49	(1.24–1.79)***	0.95	(0.78–1.16)
ICU admission	202	(2.42)	2.47	46	(2.61)	2.60	1.05	1.08	(0.78–1.49)	0.55	(0.39–0.79)***
In-hospital dialysis	73	(0.87)	0.89	10	(0.87)	0.57	0.64	0.65	(0.33–1.25)	0.40	(0.20–0.81)*
Death	137	(1.64)	1.66	34	(1.93)	1.95	1.17	1.17	(0.81–1.71)	0.41	(0.27–0.61)***

Abbreviations: IRR, Incidence rate ratio; HR, Hazard Ratio; CI, Confidence Interval; ICU, Intensive Care Unit.

^*^P value for HR < 0.05, ^**^P value for HR < 0.01, ^***^P value for HR < 0.001.

^a^Incidence rate per 100 person-year.

^b^HR were adjusted for age, sex, and comorbidities.

**Table 4:** Comparing interested outcomes risks between patients with SLE with and without vaccination stratified by age.

Age Group (years)	Nonvaccine cohort			Vaccine cohort			IRR	Adjusted HR^b^	(95% CI)
Variables	n (%)		Incidence^a^	n (%)		Incidence^a^			
Total hospitalization									
18–65	1786	(22.11)	25.71	296	(21.07)	23.95	0.93	0.87	(0.77–0.98)*
≥65	108	(38.03)	53.67	109	(30.28)	37.09	0.69	0.70	(0.54–0.92)**
Hospitalization for Pneumonia									
18–65	147	(1.82)	1.85	27	(1.92)	1.95	1.05	0.88	(0.47–1.33)
≥65	27	(9.51)	10.99	20	(5.56)	5.84	0.53	0.46	(0.25–0.83)*
Hospitalization for septicemia, bacteremia, or viremia									
18–65	137	(1.70)	1.72	14	(1.00)	1.01	0.59	0.49	(0.28–0.86)*
≥ 65	24	(8.45)	9.62	19	(5.28)	5.50	0.57	0.47	(0.25–0.87)*
Hospitalization for heart disease									
18–65	434	(5.37)	5.59	97	(6.90)	7.20	1.02	1.02	(0.82–1.28)
≥65	46	(16.20)	19.37	53	(14.72)	16.26	0.84	0.76	(0.50–1.13)
ICU admission									
18–65	171	(2.12)	2.15	23	(1.64)	1.66	0.77	0.63	(0.40–0.98)*
≥65	31	(10.91)	12.43	23	(6.39)	6.72	0.54	0.48	(0.28–0.83)**
In-hospital dialysis									
18–65	66	(0.82)	0.83	5	(0.36)	0.36	0.43	0.40	(0.20–0.81)*
≥65	7	(2.46)	2.74	5	(1.39)	1.43	0.52	0.47	(0.41–1.55)
Death									
18–65	97	(1.20)	1.21	11	(0.78)	0.79	0.65	0.52	(0.28–0.97)*
≥65	40	(14.08)	15.57	23	(6.39)	6.58	0.42	0.36	(0.21–0.71)***

Abbreviations: IRR, Incidence rate ratio; HR, Hazard Ratio; CI, Confidence Interval; ICU, Intensive Care Unit.

^*^P value for HR < 0.05, ^**^P value for HR < 0.01, ^***^P value for HR < 0.001.

^a^Incidence rate per 100 person-year.

^b^HR were adjusted for age, sex, and comorbidities.
